# Immunomodulatory Activity of Human Bone Marrow and Adipose-Derived Mesenchymal Stem Cells Prolongs Allogenic Skin Graft Survival in Nonhuman Primates

**DOI:** 10.22074/cellj.2021.6895

**Published:** 2021-03-01

**Authors:** Fattah Sotoodehnejadnematalahi, Reza Moghadasali, Mostafa Hajinasrollah, Ehsan Ehsani, Ensiyeh Hajizadeh-Saffar, Niloofar Sodeifi, Reza F. Saidi, Morteza Zarrabi, Mohammad Farzanehkhah, Bahareh Sadeghi, Hossein Baharvand, Nasser Aghdami

**Affiliations:** 1. Department of Biology, School of Basic Science, Science and Research Branch, Islamic Azad University, Tehran, Iran; 2. Department of Stem Cells and Developmental Biology, Cell Sciences Research Center, Royan Institute for Stem Cell Biology and Technology, ACECR, Tehran, Iran; 3. Department of Regenerative Medicine, Cell Sciences Research Center, Royan Institute for Stem Cell Biology and Technology, ACECR, Tehran, Iran; 4. Department of Developmental Biology, University of Science and Culture, Tehran, Iran; 5. Department of Biology, Roudehen Branch, Islamic Azad University, Roudehen, Iran; 6. Department of Andrology, Reproductive Biomedicine Research Center, Royan Institute for Reproductive Biomedicine, ACECR, Tehran, Iran; 7. Department of Surgery, Shariati Hospital, Tehran University of Medical Sciences, Tehran, Iran; 8. Royan Stem Cell Technology Company, Cord Blood Bank, Tehran, Iran

**Keywords:** Adipose, Allogenic, Bone Marrow, Immunomodulation, Mesenchymal Stem Cells, Skin

## Abstract

**Objective:**

In the present study, we examined the tolerance-inducing effects of human adipose-derived mesenchymal stem cells (hAD-MSCs) and bone marrow-derived MSCs (hBM-MSCs) on a nonhuman primate model of skin transplantation.

**Materials and Methods:**

In this experimental study, allogenic and xenogeneic of immunomodulatory properties of human AD-MSCs and BM-MSCs were evaluated by mixed lymphocyte reaction (MLR) assays. Human MSCs were obtained from BM or AD tissues (from individuals of either sex with an age range of 35 to 65 years) and intravenously injected (2×106 MSCs/kg) after allogeneic skin grafting in a nonhuman primate model. The skin sections were evaluated by H&E staining for histopathological evaluations, particularly inflammation and rejection reaction of grafts after 96 hours of cell injection. At the mRNA and protein levels, cellular mediators of inflammation, such as CD4+IL-17+ (T helper 17; Th17) and CD4+INF-γ+ (T helper 1, Th1) cells, along with CD4+FoxP3+ cells (Treg), as the mediators of immunomodulation, were measured by RT-PCR and flow cytometry analyses.

**Results:**

A significant Treg cells expansion was observed in MSCs-treated animals which reached the zenith at 24 hours and remained at a high concentration for 96 hours; however, Th1 and Th17 cells were significantly decreased. Our results showed that human MSCs significantly decrease Th1 and Th17 cell proliferation by decreasing interleukin-17 (IL-17) and interferon-γ (INF-γ) production and significantly increase Treg cell proliferation by increasing FoxP3 production. They also extend the allogenic skin graft survival in nonhuman primates. Histological evaluations showed no obvious presence of inflammatory cells or skin redness or even bulging after MSCs injection up to 96 hours, compared to the group without MSCs. There were no significant differences between hBM-MSCs and hAD-MSCs in terms of histopathological scores and inflammatory responses (P<0.05).

**Conclusion:**

It seems that MSCs could be regarded as a valuable immunomodulatory tool to reduce the use of immunosuppressive agents.

## Introduction

Mesenchymal stem cells (MSCs), as a diverse population of plastic-adherent cells, exhibit fibroblastlike morphology under the *ex vivo* culture conditions. MSCs do not express hematopoietic stem cell markers, and they are originally isolated from the bone marrow (BM), as well as other adult tissues, such as adipose tissue (1). In particular, adipose-derived MSCs (AD-MSCs) are regarded as an interesting source of MSCs, as the collection of adipose tissue is a less invasive procedure, and it is easily obtained, providing a considerable number of cells compared to BM-MSCs (2). AD-MSCs are well-known due to their immunomodulatory properties.

Because of their immunosuppressive potential, even in the absence of immunosuppressive agents, allogeneic or even xenogeneic administration of these cells into immunocompetent recipients would be feasible. AD-MSCs have been used for the treatment of a wide range of diseases, since these cells do not express major histocompatibility complex-II markers and exert immunosuppressive properties mediated by prostaglandin E2 (3, 4). In addition, preclinical and clinical investigations have shown that AD-MSCs transplantation, as allogenic agents, are able to control graft-versus-host disease (GVHD). Considering the immunosuppressive and anti-inflammatory properties of human MSCs (hMSCs), several studies suggested MSCs as an appropriate modality for cell therapy compared to other cell types (5, 6). Different *in vitro* studies showed the suppression of lymphocytes alloreactivity in mixed lymphocytes cultures mediated by a human leukocytes antigen (HLA)-independent mechanism (7, 8).

Besides, some investigations demonstrated that the intravenous administration of MSCs improves the lung, renal and neural tissue features in animal models of injury, suggesting marked paracrine effects for MSCs. Moreover MSCs can tilt the balance of pro-inflammatory and antiinflammatory cytokines in favor of anti-inflammatory cytokine production at the site of injury (9). Different studies indicated that several immune cells involved in T lymphocytes proliferation and dendritic cells maturation are suppressed by MSCs; conversely, some indicated that MSCs are able to increase the production of anti-inflammatory cytokines or induce regulatory T cell (T_regs_) activity (10-12).

It has been shown that the auto-reactive T cells play crucial roles in the secretion of cytotoxic compounds, leading to early graft rejection during the post-transplant period (13). In contrast, T_regs_ are critical cells with immunomodulatory functions (14, 15). As a subpopulation of peripheral CD4+ T cells, T_regs_ have distinct surface (e.g. CD4+CD25+) and intracellular (e.g. FoxP3+) markers and can confront T cell autoreactivity through the secretion of immunosuppressive cytokines and their surface receptors. Furthermore, they suppress antigen presentation via their inhibitory surface receptors, cytolytic function, and secretion of soluble factors (16). In view of these facts, approaches developed based on the increment of the number of Tregs, could effectively contribute to immunomodulation following skin grafting (17, 18). 

In this study, we compared the immunomodulatory properties of MSCs derived from different sources (i.e. BM and AD) in a nonhuman primate model of skin allograft.

## Materials and Methods

### Cell culture and isolation of mesenchymal stem cells from human bone marrow

In this experimental study, 100-150 ml of BM was aspirated from iliac crest of chosen patients (of either sex with an age range of 35 to 65 years) with radiologic evidence and 1.5 Tesla magnetic resonance imaging (MRI) (VB33DVision Plus; Siemens, Erlangen, Germany) of knee osteoarthritis (OA) who were selected for cell therapy under local anesthesia. Anesthesia was performed using a lidocaine solution (2%) and sedation by an intravenous injection of midazolam (0.1 mg/kg, Tehran Chemie pharmaceutical Co., Iran) and fentanyl (25-50 mg/100 mm, Aburaihan pharmaceutical Co., Iran). BM was collected in a centrifuge tube (50 ml, TPP, Switzerland), containing anti-coagulant (Heparin, Rotexmedica, Germany; 300 μl Rotexmedica for 50 ml of BM). The aspirated BM was diluted at a ratio of 1:1 with α-MEM medium (Gibco, USA); then, layered very gently onto Lymphodex solution (gravity: 1.077-1.080; Inno-Train Diagnostik, Germany) and centrifuged (Hettich Universal 320, Germany) at 1400 rpm for 30 minutes to collect mononuclear cells (MNCs). MNCs were then re-suspended in 5ml of α-MEM (Gibco, USA) supplemented with 10% fetal bovine serum (FBS, Hyclone, USA), 1% L-glutamine (L-glu, Gibco, USA), and 1% penicillin/streptomycin (pen/ strep, Gibco, USA). The culture was maintained at 37˚C in a humidified atmosphere (Labotect CO_2_-incubator, Germany), containing 95% air and 5% CO_2_ and passaged every 3 days. Fibroblast-like MSCs became ready for the characterization after the third passage. 

All animal care, experimental, and transplantation processes and postoperative euthanasia were performed in strict accordance with the ethical principles of the NIH Guide for the Care and Use of Laboratory Animals (NIH Publication No. 85-23, revised 2010) following the approval of the Institutional Review Board and Institutional Ethics Committee of Royan Institute (approval No. EC.92.1005).

### Cell culture and isolation of mesenchymal stem cells from human adipose tissue

Adipose tissue was first isolated by liposuction from abdominal subcutaneous fat of individuals (of either sex with an age range of 35 to 65 years) and then, transferred into a sterile tube (50 ml, TPP, Switzerland), containing phosphate-buffered saline (PBS, Gibco, USA) and 1% pen/strep (Gibco, USA). The tube was kept on the ice. The adipose tissue was washed several times with sterile water to remove red blood cells. Then, the tissue (which is normally between 150-250 ml) was sectioned into smaller pieces and 0.075% collagens I (Sigma, USA) was added for digestion. The tissues were placed in an incubator (with 5% CO_2_ at 37˚C) for 2 hours, while spinning every 15 minutes. After 2 hours, α-MEM (Gibco, USA, at twice concentration of the enzyme) was added to the tube to neutralize the enzyme by pipetting up and down to release the cells from adipose tissue. Then, the sample was centrifuged at 1500 rpm for 5 minutes, and the pellet (stromal vascular fraction) was diluted in 4-5 ml of α-MEM (Gibco, USA). Afterward, the suspension was passed through a Mesh filter (Falcon, UK). The cells were transferred to a 25T flask (TPP, Switzerland), containing α-MEM supplemented with 10% FBS (Hyclone, USA), 1% pen/strep (Gibco, USA), and 1% L-glu (Gibco, USA) and kept in an incubator (with 5% CO_2_ at 37˚C). The medium was replaced with a fresh medium every four days until reached 90% confluency. The cell culture was continued until the third passage.

### Analysis of the cell surface markers of human bone marrow-derived mesenchymal stem cells and adiposederived mesenchymal stem cells

Surface markers of human bone marrow-derived mesenchymal stem cells (hBM-MSCs) and adiposederived mesenchymal stem cells (hAD-MSCs) were analyzed using fluorescence-activated cell sorting (FACS, BD Pharmingen, USA). For immunophenotyping, hBMMSCs and hAD-MSCs were dissociated in 0.05% trypsinEDTA (Gibco, USA) and washed in PBS (Gibco, USA) supplemented with 1% heat-inactivated FBS (Hyclone, USA) and 2 mM EDTA (Merck, Darmstadt, Germany). Next, 4-5×10^5^ cells were incubated with primary antibodies for surface markers for two hours and with the secondary antibodies for 30 minutes, both done at 4˚C. Surface markers that were analyzed included CD44, CD73 (BD Pharmingen, USA), CD105 (R&D Systems Inc, Minneapolis, MN, USA) conjugated with phycoerythrin (PE)-Mouse IgG1k (BD PharmingenTM, Cat NO: 551436) and CD90 (Dako, Glostrup, Denmark) conjugated with fluorescein isothiocyanate (FITC)-Mouse IgG2b (Millipore, Cat NO: MABC006F), which were supposed to be expressed by fully differentiated MSCs, as well as CD34 and CD45 (BD Pharmingen) conjugated with FITC, which are markers of hematopoietic stem cells (HSCs), and they are not expressed on MSCs. In all experiments, controls were stained with appropriate isotype-matched antibodies. The flow cytometry analysis was performed triplicate using a BD FACS Calibur Flow Cytometer (BD Biosciences, Franklin Lakes, NJ, USA). Data were analyzed by WinMDI 12.9 software (freeware from Joe Trotter, The Scripps Research Institute, La Jolla, CA, USA).

### Lineage differentiation for characterization of human bone marrow-derived mesenchymal stem cells and adipose-derived mesenchymal stem cells

For further characterizations, osteogenic and adipogenic differentiations of hBM- and hAD-MSCs were induced using the following protocol. In this stage, 1×10^4^ cells per well (TPP, Switzerland) were seeded in 6-well plates and treated with conductive medium for 21 days. The media of wells were changed every three days. At 50% confluency, the medium was supplemented with 0.5 µM ascorbic acid-2-phosphate (Sigma-Aldrich, USA), 1 µM dexamethasone (Stem Cell Technologies, Canada), and 10 mM β-glycerophosphate (Sigma-Aldrich, USA) for osteogenic induction. The cells were analyzed for mineralization using alizarin red (Sigma-Aldrich, USA) staining. To induce adipogenic differentiation, cells were incubated with complete medium including 50 µg/ml indomethacin (Sigma-Aldrich, USA), 100 nM dexamethasone (Sigma-Aldrich, USA), insulin (SigmaAldrich, USA), and 3-isobutyl-1-methylxanthine (SigmaAldrich, USA). Finally, the cells were analyzed for lipid content by oil-red (Sigma-Aldrich, USA) staining. 

### Non-proliferating lymphocytes analysis using cell proliferation assay

**Fig.1:**
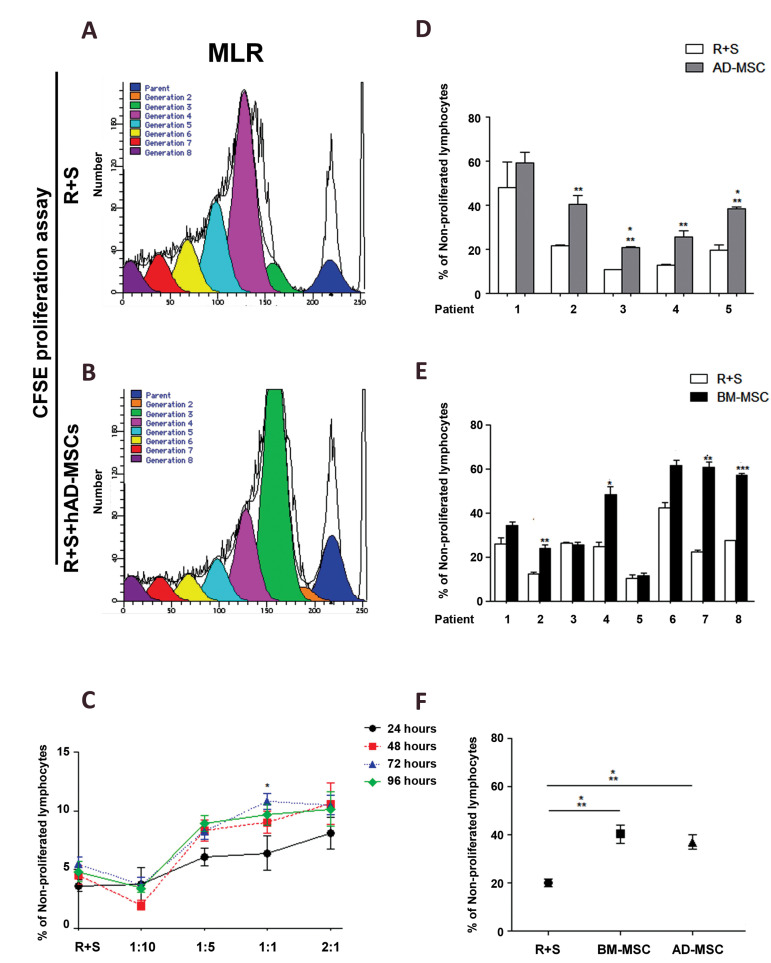
Allogenic immunomodulatory properties of *in vitro* expanded hAD-MSCs obtained from the aspirate of adipose tissue. **A-C.** Immunomodulatory properties of hAD-MSCs were assessed in the MLR medium following co-culture of hAD-MSCs and responder (R) and stimulator (S) human T cells (R+S+AD-MSCs) at different ratios (1:10, 1:5, 1:1, and 2:1) and different time periods (24, 48, 72, and 96 hours). The cell ratio of 1:1 and culture period of 72 hours were selected as the optimal conditions. The comparison of the immunomodulatory properties of **D.** hAD-MSCs (n=5 patients) and **E.** hBM-MSCs (n=8 patients), was made under the optimal conditions in the MLR medium. No significant difference was observed among R+S and R+S+BM-MSCs (patients 1, 3, and 5), and R+S+AD-MSCs (patient 1). **F.** Also, no significant difference was found between hAD-MSCs and hBM-MSCs. Data are presented as the mean ± standard deviation. *; P<0.05, **; P<0.01, ***; P<0.001, hAD-MSCs; Human adipose-derived mesenchymal stem cells, hBM-MSCs; Human bone marrow-derived mesenchymal stem cells. MLR; Mixed lymphocyte reaction, BM-MSCs; Bone marrow-derived mesenchymal stem cells, and AD-MSCs; Adipose-derived mesenchymal stem cells.

Responder T cells were first labeled with carboxyfluorescein diacetate succinimidyl ester (CFSE) (Invitrogen, USA). For this purpose, responder T cells were labeled with 1 µM of CFSE for 15 minutes at 37˚C in PBS (Gibco, USA) supplemented with 0.1% bovine serum albumin (BSA, Sigma-Aldrich, USA). Cells were washed twice with PBS (Gibco, USA) + 1% FBS (Hyclone, USA), re-suspended in media+10% FBS (Hyclone, USA), and incubated at room temperature for further 10 minutes. Then, the cells were collected and analyzed by flow cytometry. Immunomodulatory properties of hAD-MSCs and hBM-MSCs were assessed in the MLR medium, including responder (R) and stimulator (S) human T cells (R+S+AD-MSCs), and CFSE-labeled responder T cells were added to cultured MSCs at different ratios of 1:10, 1:5, 1:1 and 2:1 co-culture ratio of MSCs to responder T cells for 24, 48, 72, and 96 hours (Fig .1A-C).

### Immunosuppressive activity of mesenchymal stem cells in mixed lymphocyte reaction 

For the assessment of immunomodulatory properties of allogenic hAD-MSCs and hBM-MSCs, MNCs were isolated from the peripheral blood of humans. For the evaluation of immunomodulatory properties of xenogeneic hAD-MSCs and hBM-MSCs, MNCs were isolated from the peripheral blood of monkeys by the Lymphodex solution (gravity: 1.077-1.080; Inno-Train Diagnostik, Germany) and used as stimulators and responders. For peripheral blood sampling from primates, the tibial vein was used. Then, the skin over the venipuncture site was sterilized using alcohol (70%). For blood sampling, a needle (1.2-2.0 mm) and a syringe (2.5-10 mL) were used. Then, the needle was withdrawn, and the area over the vein was put under pressure for at least one minute, to avoid hematoma formation. Then, peripheral blood (5 ml) was collected under heparin (Heparin, Rotexmedica, Germany) and MNCs were isolated from heparinized blood by gradient centrifugation as stimulators and responders. The stimulators, but not the responders, were treated with mitomycin C (MMC, M0503, Sigma-Aldrich, USA) (50 μg/ml at 37˚C for 1 hour). The stimulators (5×10^5^ /well) and responders (2.5×10^5^ /well) as the experimental groups were loaded into a 96-well plate and MMC-treated MSCs (2.5×10^5^ /well) were added. After 5-day routine culture, the MTT solution (5 mg/ml PBS) (Sigma-Aldrich, USA) was added, and the cells were incubated for 4 hours at 37˚C. Afterwards, the MTT solution was removed, and 200 µl DMSO (WAK-Chemie Medical, Germany) was added. The extinction of the solution was measured at 570 nm using a Multiskan Bichromatic microplate reader (Labsystems, Helsinki, Finland) to assay immunosuppressive activity (Figes.1D-F, 2A-C).

### Human mesenchymal stem cells transplantation into healthy monkeys

After washing hAD-MSCs and BM-MSCs twice with PBS (Gibco, USA)+1% FBS (Hyclone, USA), the cells were re-suspended in media supplemented with 10% FBS (Hyclone, USA) and incubated at room temperature for further 10 minutes. Then, rhesus monkeys were intravenously injected with approximately 2×10^6^ MSCs/ kg. The blood specimens as heparinized were collected at certain time points (i.e. 6, 12, 24, 48,72, and 96 hours) and assessed by real-time polymerase chain reaction (RTPCR) and flow cytometry. 

### Real-time polymerase chain reaction

cDNA was synthesized using 100 ng total RNA by SuperScript™ III Reverse Transcriptase (Life Technologies, USA) and amplified by ExTaq (Takara, Japan). RT-PCR was performed using Platinum SYBR Green qPCR SuperMix-UDG plus ROX (Invitrogen, USA), according to the manufacturer’s instructions in an ABI7300 RT-PCR System (Applied Biosystems, USA). Primer sequences were as follows:

GAPDH-

F: 5´-CTCATTTCCTGGTATGACAACGA-3´ 

R: 5´-CTTCCTCTTGTGCTCTTGCT-3´

FoxP3-

F: 5´-CCAGCCATGATCAGCCTCAC-3´

R: 5´-CCGAAAGGGTGCTGTCCTTC-3´

INF-γ-

F: 5´-GGTTCTCTTGGCTGTTACTG-3´

R: 5´-TCTTTTGGATGCTCTGGTCA-3´

IL-17-

F: 5´-AACCGATCCACCTCACCTTG-3´

R: 5´-CCCACGGACACCAGTATCTT-3´

Real-time PCR data were analyzed by an ABI PRISM 7500 RT-PCR program. RT-PCR program included polymerase activation and initial denaturation (95˚C, 10 minutes), denaturation (95˚C, 10 minutes), annealing and extension (60˚C, 60 seconds) for 40 cycles. All of the absolute data were normalized against a housekeeping gene (*GAPDH*) and control group, including embryonic stem cells (ESCs) and mouse embryonic fibroblast (MEF) using the ∆∆Ct method. The assay was run in triplicate to obtain gene expression data. 

### Intracellular staining for flow cytometry 

Single T cell suspensions (0.1×10^6^ cells) were washed with BD Perm/Wash Buffer (BD, London, UK). After washing, 200 μl BD Cytofix/Cytoperm solution (BD, USA) was added to each cell pellet, and the cells were incubated for 20–30 minutes at 4˚C. The cells were then washed twice with BD Perm/Wash Buffer and incubated in PBS (pH=7.4) (Gibco, USA), containing 5% BSA (Sigma-Aldrich, USA) for 10-15 minutes at room temperature. After washing with BD Perm/Wash Buffer, the cells were aliquoted into tubes and then treated with a conjugated antibody based on the manufacturer’s protocol (i.e., incubated for 30-45 minutes at 4˚C in dark). Th-1 and Th-17 cells were examined for the expression of intracellular cytokines IL-17 (eBioscience, USA), IFN-γ (BD Bioscience, USA), Foxp3 (Biolegend, San Diego, CA, USA), and CD25 (BD Bioscience) conjugated with PE-labelled mouse anti-human antibodies and CD4 conjugated with FITC-labelled mouse anti-human antibodies, as well as PE-conjugated mouse IgG1 isotype control (BD Bioscience) by cell staining. All samples were analyzed by a flow cytometer (BD FACSCalibur^TM^, USA) and FlowJo software.

### ELISA assay for cytokine production

As the control and responder T cells to immunosuppressive factors, at the protein level, T cells isolated from rhesus after allogenic skin grafting and before hAD-MSCs transplantation, were exposed to TGF-β (10 and 20 ng/ ml) (Sigma-Aldrich, USA) as an immunosuppressive factor, and the percentage of IL17 and IFN-γ were decreased. The supernatant of wells, containing T cells treated with TGF-β (10 and 20 ng/ml) was evaluated for IL-17 and IFN-γ, as immunomodulatory cytokines, using a commercially available enzyme-linked immunosorbent assay (ELISA; eBioscience, USA), according to the manufacturer’s protocol.

### Rhesus model of skin allograft

Eight healthy male rhesus monkeys (weighing 3-5 kg) were used for the induction of a model of skin allograft. Monkeys were gifted from the Royan Institute Primate Research Center. In this study, we used the minimum possible number of animals. Animals were housed individually in latticed cages (2×2×2 m^3^), and they had free access to food and water throughout the study. The cages were equipped by door handles for animal visit, sampling, and injection. The cage floor was made of PVC (Polyvinyl chloride) pipes for the prevention of bedsores. Also, these pipes allow the drainage of urine and stool. Also, they were assessed for tuberculosis, simian immunodeficiency virus, herpes viruses A and B, and hepatitis viruses A and B. To ensure that monkeys receiving transplanted skin are not genetically identical by a chance, ABO grouping, HLA typing (HLA-ABC-FITC and HLA-DR-PE, eBioscience, USA), RBC cross-match (Table 1), and mixed-lymphocyte reaction test were performed before transplantation on donor and recipient lymphocytes for tissue typing before transplantation. Then, eight monkeys were divided into two groups. In each group, four monkeys underwent heterotopic cross skin grafts transplantation (4×4 cm skin patch) pairwise under inhaled anesthesia Generally, donor skin grafts are typically taken from the back wall and implanted on the back of the recipient site to reduce the probability of animal picking at the graft site. It is essential to remove cutaneous fat tissue from the skin graft before transplantation; however subcutaneous tissue and microvasculature are not removed from the recipient site. For one pair, one monkey received a total of 2×10^6^ hBM-MSCs/kg (test), while another received no treatment (control). For another pair, oe monkey received a total of 2×10^6^ hAD-MSCs/kg (test), whereas another received no treatment (control). The cells were intravenously transplanted into rhesus monkeys on day 0. Allograft rejection was monitored macroscopically by graft peripheral redness and bulging, and histological evaluations of rejection were carried out microscopically on the skin biopsy at appropriate time points.

### Histopathological analyses and qualitative evaluations of inflammation and rejection

Histopathological analyses were performed 96 hours after allogenic skin grafting. Tissues were washed twice with PBS (Gibco, USA) and then fixed with 4% paraformaldehyde (Sigma-Aldrich, USA) for 24 hours at 4˚C. Afterwards, the tissues were dehydrated through a series of graded alcohol solutions and xylol and then embedded in paraffin. The paraffinembedded tissues were sectioned into 5-μm thick sections, mounted on poly-l lysine (Sigma-Aldrich, P1524, USA)-coated glass slides and placed in an oven at 60˚C for 12 hours. Next, they were deparaffinized and dewaxed in xylene, stained with hematoxylin and eosin (H&E) and observed under a light microscope. To assess the presence of rejection, H&E-stained sections were examined and scored for inflammation, as previously described. Briefly, inflammatory cells, including polymorphonuclear leukocytes (PMNs), non-phagocytic cells, and phagocytic cells were scored based on the following scales: 0: No cell, 1: 1 to 5 cells per high-power field (hpf=400x), 2: 6 to 25 cells per high-power field (hpf=400x), 3: 26 to 50 cells per high-power field, 4: 51 to 75 cells per high-power field, 5: 76 to 100 cells per high power-field and 6: Over 100 cells per high-power field. Also, epidermis was scored as follows: 0: Normal, 1: Completely healed, 2: Healed but thin, 3: Ulcerative but healing and 4: Completely ulcerative or destroyed. Since we did not have more than two animals, histopathological scores were not statistically analyzed, and the evaluations of inflammation and rejection were only presented as a situational report on MNCs infiltration, presence and absence of phagocytic and non- phagocytic cells, and epidermis destruction or healing. 

### *In vivo* analysis of Th1, Th17 and T reg populations

To test the immunomodulatory effect of hMSCs on Th1, Th17 and Treg population and related cytokines production, *in vivo* assays were carried out as follows; a total of 2×10^6^ of hBM-MSCs/kg or hAD-MSCs/kg were intravenously injected to rhesus monkeys on day of skin grafting. Peripheral blood MNCs (PBMNCs) were isolated from heparinized blood by gradient centrifugation at appropriate time points (24, 48, 72, and 96 hours) following transplantation and then, Th1 markers (antiCD4 and anti-IFN-γ), Th17 markers (anti-CD4 and antiIL-17) and Treg markers (anti-CD4 and anti-FoxP3) were analyzed using flow cytometry. 

### *In vivo* cytokine assessments

To determine the mRNA levels of cytokines released by T cells, some MNCs isolated in the above-noted experiment, were analyzed for mRNA level of cytokines, such as IFN-γ, IL-17, and FoxP3, which are released by Th1, Th17 and Treg, respectively. 

### Statistical analysis

Because of having a small sample size, histopathological evaluations were reported as descriptive and each report was confirmed or rejected with additional experiments. Cellular data were measurable as statistical comparisons, and the analysis was conducted by the GraphPad Prism version 7.03 (GraphPad Software, Inc., USA). Data are presented as mean ± standard deviation (SD) of the mean for a minimum of three measurements at each time point. Statistical analysis was performed using one-way ANOVA to evaluate significant differences between groups at P<0.05.

**Table 1:** ABO grouping, RBC cross-matching, and HLA typing of monkeys


Blood typing	Monkey code	ABO/Rh	RBC	HLA																			
			cross-match scores	A	B														DR				
				14	5	7	18	19	23	24	34	39	43	44	45	46	47	48	10	14	16	17	18

No cell	2005	B-	+2	*	-	*	-	-	*	*	*	*	*	-	-	-	*	*	-	-	-	-	-
BM-MSCs	2010	B-	+2	*	*	*	*	-	*	*	-	*	*	*	-	-	*	*	-	*	*	*	-
No cell	2017	B-	+2	*	*	*	-	-	-	*	-	*	-	-	*	-	*	*	-	-	-	-	-
AD-MSCs	2011	B-	+2	*	*	*	-	*	*	*	-	*	-	*	-	*	*	*	*	-	-	-	*


47: HLA-B and 48: HLA-B were considered controls., ABO blood group, RBC; Red blood cells, HLA; Human leukocyte antigen, BM-MSCs; Bone marrow-derived mesenchymal stem cells, and AD-MSCs; Adipose-derived mesenchymal stem cells.

## Results

### Characterization of human adipose-derived mesenchymal stem cells and bone marrow-derived mesenchymal stem cells

hAD-MSCs and BM-MSCs obtained from Royan Stem Cell Bank (RSCB) showed a fibroblastic spindleshaped morphology after two weeks of culture. To verify differentiation capacity, hAD-MSCs and BM-MSCs were differentiated into adipocyte and osteocyte lineages in specific induction media. Oil red and alizarin red dye were used to examine adipogenic and osteogenic differentiation capacity, respectively. Immunophenotypic characterization of MSCs was performed by a Flow cytometer (BD FACSCalibur^TM^) and FlowJo software. MSCs (hAD vs. hBM) were positive for CD44 (83 vs. 99.54%, respectively), CD73 (89 vs. 94.06%, respectively), CD90 (94 vs. 88.91%, respectively) and CD105 (77 vs. 96.74%, respectively), as mesenchymal stem cell markers. The results showed that hAD-MSCs and hBM-MSCs were not contaminated by hematopoietic cell lineages [i.e. cells were CD34 and CD45 negative) (Fig.S1, See Supplementary Online Information at www.celljournal.org]. 

### *In vitro* immunosuppressive capacity of human adipose-derived mesenchymal stem cells and bone marrow-derived mesenchymal stem cells

Allogenic immunomodulatory properties of hAD-MSCs were assessed in the MLR medium, containing responder (R) and stimulator (S) human T cells (R+S+AD-MSCs) at different ratios of 1:10, 1:5, 1:1, and 2:1 at various time periods (24, 48, 72, and 96 hours). The cell ratio of 1:1 and culture period of 72 hours, showed optimal results (Fig .1A-C). Immunomodulatory properties of hAD-MSCs (n=5 patients) (Fig .1D) and hBMMSCs (n=8 patients) (Fig .1E) were assessed at optimal ratios in the MLR medium. There were no significant differences among R+S and R+S+BM-MSCs (patients 1, 3, and 5), and R+S+AD-MSCs (patient 1). So, these patients were excluded at later stages. There were no significant differences between hAD-MSCs and hBM-MSCs (Fig .1F).

**Fig.2:**
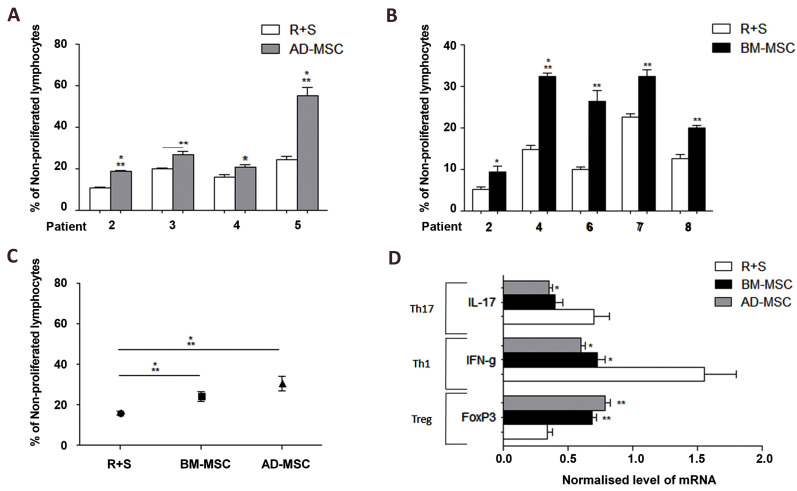
Xenogeneic immunomodulatory properties of hAD-MSCs and hBM-MSCs. **A.** Xenogeneic immunomodulatory properties of human AD-MSCs and **B.** BMMSCs were evaluated in the MLR medium following co-culture of hAD-MSCs and responder (R) and stimulator (S) monkey T cell (R+S+AD-MSCs) under the optimal conditions (i.e., at the ratio of 1:1 for 72 hours). The comparison of the immunomodulatory properties of hAD-MSCs (patients 2, 3, 4, and 5) and hBM-MSCs (patients 2, 4, 6, 7 and 8) was made under the optimal conditions in the MLR medium. **C.** Significant differences were found among R+S and R+S+BM-MSCs, and R+S+AD-MSCs in all groups. Also, no significant difference was observed in xenogeneic immunomodulatory properties between hBM-MSCs and hAD-MSCs. **D. ***In vitro* immunomodulatory effects of hAD-MSCs and hBM-MSCs on rhesus T cells subset were assessed. There were significant differences in the mRNA level of *IL-17* (Th17), *IFN-γ* (Th1) and *Treg* (FoxP3) among hBM-MSCs and hAD-MSCs, and R+S. Data are presented as the mean ± standard deviation. *; P<0.05, **; P<0.01, ***; P<0.001, hAD-MSCs; Human adipose-derived mesenchymal stem cells, and hBM-MSCs; Human bone marrow-derived mesenchymal stem cells.

Xenogeneic immunomodulatory properties of human ADMSCs (Fig .2A) and BM-MSCs (Fig .2B) were evaluated in the MLR medium by co-culturing hAD-MSCs, responder (R) and stimulator (S) monkey T cells (R+S+AD-MSCs) under optimal conditions (i.e., at the ratio of 1:1 for 72 hours). Immunomodulatory properties of hAD-MSCs (from patients 2, 3, 4, and 5) and hBM-MSCs (from patients 2, 4, 6, 7 and 8) were evaluated under optimal conditions in the MLR medium. There were significant differences among R+S, R+S+BM-MSCs, and R+S+AD-MSCs in all groups. However, there was no significant difference in xenogeneic immunomodulatory properties when comparing hBM-MSCs and hAD-MSCs (Fig .2C). *In vitro* immunomodulatory effects of hAD-MSCs and hBM-MSCs on rhesus T cells subset were assessed. Significant differences were observed in the mRNA level of *IL-17* (Th17), *IFN-γ* (Th1) and *Treg* (FoxP3) between hBM-MSCs and hAD-MSCs, as compared to R+S alone (Fig .2D).

### *In vivo* immunomodulatory effect of hMSCs on T cells of healthy recipient monkeys

**Fig.3:**
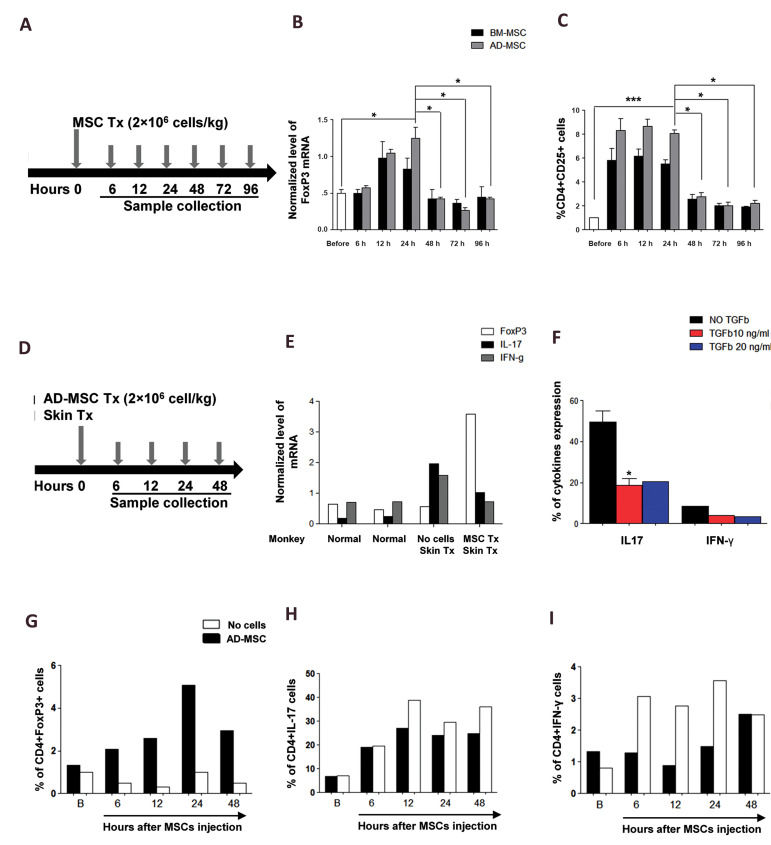
Immunomodulatory effects of hAD-MSCs and hBM-MSCs transplantation on T cell subsets in a healthy monkey recipient and immunomodulatory effects of hAD-MSCs transplantation on rhesus T cell subsets after skin grafting. **A.** A schematic overview of *in vivo* cell transplantation in a healthy monkey recipient. **B, C.** No significant differences in xenogeneic immunomodulatory properties were found between hBM-MSCs and hAD-MSCs in a healthy monkey recipient, 6-96 hours after cell transplantation. However, a significant difference was found in xenogeneic immunomodulatory properties when comparing hBM-MSCs and hAD-MSCs in terms of Foxp3+ T cells and CD4+CD25+ T cells (*P<0.05), 24-96 hours after cell transplantation. Also, a significant difference was observed in xenogeneic immunomodulatory properties between hBM-MSCs and hAD-MSCs in terms of Foxp3+ T cells (*P<0.05) and CD4+CD25+ T cells (***P<0.001), before 24 hours of cell transplantation. **D.** Schematic overview of *in vivo* hAD-MSCs transplantation with skin graft. **E.** Immunomodulatory effect of hAD-MSCs transplantation on monkey T cells subsets 48 hours after skin grafting in a monkey model. **F.** As a control, at the protein level, T cells of rhesus after skin graft and before hAD-MSCs transplantation were exposed to TGF-β (10 and 20 ng/ml) as an immunosuppressive factor, and the percentage of IL17 and IFN-γ were decreased. **G-I.** At the protein level, after hAD-MSCs IV transplantation, 6-48 hours after cell transplantation, the percentage of CD4+IL-17+ and CD4+INF-γ+ cells, as the cellular mediators of inflammation, was decreased, while the number of CD4+FoxP3+ cells, as the mediators of immunomodulation, was increased. Data are presented as the mean ± standard deviation. *; P<0.05, ***; P<0.001, hAD-MSCs; Human adipose-derived mesenchymal stem cells, and hBMMSCs; Human bone marrow-derived mesenchymal stem cells. h; Hour; IL; Interleukin ,and IFN; Interferon.

hAD-MSCs and hBM-MSCs (approximately 2×10^6^ MSCs/animal) were intravenously injected to healthy rhesus monkeys (Fig .3A). The blood samples were assessed for FoxP3+ T cells (by RT-PCR, Fig .3B) and CD4+CD25+ T cells (by flow cytometry) (Fig .3C) at appropriate time points (6, 12, 24, 48, 72, and 96 hours). No significant difference was found in xenogeneic immunomodulatory properties between hBM-MSCs and hAD-MSCs in a healthy recipient monkey, 6-96 hours after MSC transplantation. However, there was a significant difference in xenogeneic immunomodulatory properties between hBM-MSCs and hAD-MSCs in Foxp3+ T cells and CD4+CD25+ T cells (P<0.05), 24-96 hours after MSC transplantation. Also, there was a significant difference in xenogeneic immunomodulatory properties between hBM-MSCs and hAD-MSCs in Foxp3+ T cells (P<0.05) and CD4+CD25+ T cells (P<0.001), before 24 hours of MSC transplantation (Fig .3B, C).

### Skin grafting and immunomodulatory effects of human adipose-derived mesenchymal stem cells

Immunomodulatory effects of hAD-MSCs were evaluated on T cell subsets after skin grafting at different time points (6, 12, 24, and 48 hours, Fig .3D). Foxp3, IL-17 and INF-γ expression levels were compared after grafting hAD-MSCs (48 hours) and the skin (Fig .3E). At the protein level, as a control, T cells of rhesus after allogenic skin grafting and before hAD-MSCs transplantation were treated with TGF-β (10 and 20 ng/ml) as a immunosuppressive factor, and the percentages of IL17 and IFN-γ were decreased (Fig .3F). Results showed that after hAD-MSCs injection, CD4+IL-17+ (Th17) and CD4+INF-γ+ (Th1) cells were decreased, while CD4+FoxP3+ cells (Treg) were increased (Fig .3G-I).

### Comparative immunomodulatory effect of human adipose-derived mesenchymal stem cells and bone marrow-derived mesenchymal stem cells after skin grafting 

**Fig.4:**
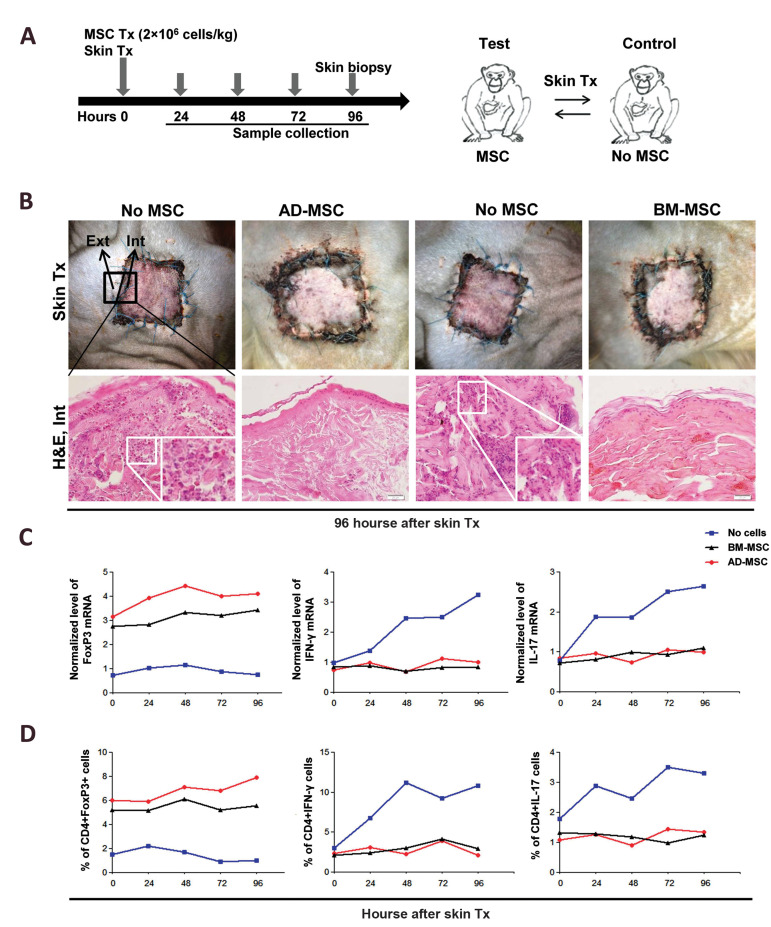
Immunomodulatory effect of human adipose-derived mesenchymal stem cells (hAD-MSCs) and human bone marrow-derived mesenchymal stem cells (hBM-MSCs) transplantation on rhesus T cell subsets after allogenic skin grafting. **A.** A schematic overview of *in vivo* cell transplantation with skin grafting. Immunomodulatory effects of hAD-MSCs and hBM-MSCs transplantation on monkey T cells subsets 24, 48, 72, and 96 hours after skin grafting in a monkey model. **B.** Inflammatory features, such as redness and bulging can be detected around skin graft area without cell transplantation. Visual inspection and histopathological analysis of transplanted tissues showed mild inflammation around allogenic skin graft after cell transplantation compared with monkeys receiving no cell transplantation and showed severe inflammation. **C.** At the mRNA level, after IV transplantation of hBM-MSCs and hADMSCs, the percentages of Th1 and Th17, as the cellular mediators of inflammation, were decreased, while the percentage of Treg, as mediators of immunomodulation, was increased. **D.** Also, at the protein level, after IV transplantation of hBM-MSCs and hAD-MSCs, the percentages of Th1 and Th17 were decreased, while the number of Tregs was increased.

HAD-MSCs and hBM-MSCs (2×10^6 ^MSCs/kg) were intravenously transplanted into rhesus monkey on the day of skin grafting (Fig .4A). The skin sections were analyzed by H&E staining for inflammation and rejection, 96 hours after transplantation. Histological assessments showed no trace of inflammation and exhibited redness or bulging in the internal part of skin biopsies after MSCs transplantation up to 96 hours, compared to the group without MSCs (Fig .4B). However, there were no significant differences between hAD-MSCs and hBM-MSCs in histopathological scores in terms of PMNs, non-phagocytic cells, and phagocytic cells counts and destroyed epidermis (Table.2). Also, RT-PCR and flow cytometry were used to detect Th1 (ani-CD4 and anti-IFN-γ), Th17 (anti-CD4 and anti-IL-17) and T regulatory markers (anti-CD4 and anti-FoxP3). At the mRNA level, after the intravenous transplantation of MSCs, the percentages of Th1 and Th17 cells were reduced, while the percentage of Treg cells was increased (Fig .4C). Also, at the protein level, after intravenous transplantation of MSCs, the percentages of Th1 and Th17 cells were decreased, while Treg cells were increased (Fig .4D). These results showed that MSCs have immunomodulatory properties. However, there was a pilot *in vivo* evaluation with a small sample size of monkeys because of some limitations in time and cost for the proof-of-concept of immunomodulatory properties of MSCs. So, we could not draw any significant conclusion on the efficacy due to the experiment design, including limited sample size, lack of control group, and single-dose infusion. It seems that we need more animal samples, skin biopsies and other tissue samples to perform the statistical analysis of immunomodulatory properties of MSCs after transplantation in future studies.

**Table 2:** Inflammatory cells and epidermal healing scoring after skin transplantation with and without MSCs up to 96 hours


Groups	Site	Inflammatory cells			Epidermis
					Destroyed (0 to 4)
		PMNs (0 to 6)	Non-Phagocytic cells (0 to 6)	Phagocytic cells (0 to 6)	

No cell	Ext	0	1	1	0
	Int	5	N/A	N/A	3
BM-MSCs	Ext	0	2	1	0
	Int	6	N/A	N/A	4
No cell	Ext	6	1	1	1
	Int	3	1	0	2
AD-MSCs	Ext	0	2	2	1
	Int	4	N/A	N/A	3


Ext; External biopsy, Int; Internal biopsy, N/A; Not applicable (because of the presence of acute inflammation), PMN; Polymorphonuclear cells, Nonphagocytic cells; Lymphocytes and plasma cells, BM-MSCs; Bone marrow-derived mesenchymal stem cells, and AD-MSCs; Adipose-derived mesenchymal stem cells.

## Discussion

Several experiments showed that the beneficial paracrine effects of MSCs transplantation are stronger than their differentiation ability (19). MSCs are now known to have potent anti-inflammatory and immunomodulatory properties besides their regenerative capacities (20, 21). The immunomodulatory potential of hBM-MSCs and hAD-MSCs has led to their application against various inflammatory and auto-immune disorders as well as organ transplantation (22). In this regard, studies showed that autologous or allogenic MSCs could suppress the proliferation of both CD4+ and CD8+ T lymphocytes, which were stimulated by mitogens or specific antigens (23) via mechanisms, which are not limited to major histocompatibility complex [MHC, (7)]. In addition, MSCs affect other T cells functions, including a decrement in proinflammatory factors, such as IFN- γ, IL-2, and TNFα, along with an increment in the secretion of IL-4 and IL10, which are well-known for their anti-inflammatory effects (24). Although several studies have reported the immunosuppressive effects of MSCs on other immune cells, such as B cells (25), neutrophil cells (26), natural killer (NK) cells (27) and dendritic cells [DC, (28)]. *in vitro* and *in vivo* studies highlighted the increased generation of CD4+CD25+ T regulatory cells as a critical part of MSCs immunosuppressive effects (21, 29).

MSCs act via cell-cell contact and releasing soluble factors, such as transforming growth factor (TGF)-β, hepatocyte growth factor (HGF) (30), prostaglandin E2 [PGE2 , (31)]. indoleamine-2,3-dioxygenase, inducible nitric-oxide synthase [iONS, (32)]. and IL-10 (33), which promote lymphocytes suppression, and they were reported to be potentially responsible for immunomodulatory effects of MSCs. In general, MSCs isolated from various sources, such as the bone marrow, adipose tissue, and Wharton’s jelly have shown somehow similar suppressive effects on the proliferation of CD4+ and CD8+ T-cell populations (34). In this study, allogenic and xenogeneic immunomodulatory properties of hAD-MSCs and hBM-MSCs were confirmed *in vitro* on human and monkey T cell subsets before transplantation. Also, a 1:1 cell ratio and a culture period of 72 hours showed the optimal results for immunomodulatory properties and selected for next analyses.

T helper cells (Th) are CD4+ subset of T cells that recognize cell surface proteins presented by MHC. Their differentiation into Th1, Th2, and Th17 cells depends on cytokine environment around the site of the antigen presentation (35). When CD4+ T cells are induced in the presence of IL-12 and IFN-γ, they shift toward Th1 phenotype. IFN-γ is a pivotal cytokine produced by Th1 cells. Th1 cells promote the activation and recruitment of macrophages to the inflammation site and induce the removal of intracellular pathogens and delayed-type hypersensitivity (DTH) reactions by activating cellular immunity responses (36). Another pro-inflammatory subset of Th cells is Th17 cell, an effector phenotype characterized by preferential secretion of IL-17A (IL-17), while expressing other cytokines, including IL-17 F, IL-21, and IL-22. Although most of recent studies indicated that MSCs are able to suppress Th17 cell-mediated immune responses via different mechanisms, some experiments showed Th17 cell-promoting effects on MSCs (37).

Treg is a subset of CD4+ T cells with potent suppressive functions necessary for the prevention of autoimmune conditions and reduction of inflammatory reactions via cell-cell contact and secretion of soluble factors. These cells are generally characterized by the expression of a surface marker CD25 (IL-2 receptor alpha chain) and the intracellular marker FOXP3. Treg could downregulate the activation of inflammatory Th cells subtypes (i.e. Th1 and Th17), just like other inflammatory cells. As indicated in several *in vitro* and *in vivo* studies, MSCs could increase the number and functionality of Treg cells (17, 21, 38-40). 

In our study, immune modulatory effect of hADMSCs and hBM-MSCs transplantation on monkey T cell subsets, 96 hours after allogenic skin grafting, was assessed in a monkey model. Inflammatory features, such as redness and bulging were observed around allogenic skin graft area in the absence of hADMSCs and hBM-MSCs transplantation. Also, visual inspection and histopathological analysis showed mild inflammation around allogenic skin graft after cell transplantation, compared with monkeys receiving no cell transplantation and showed high inflammation. At the mRNA and protein levels, after the intravenous transplantation of hBM-MSCs and hAD-MSCs, the percentages of CD4+IL-17+ (Th17) and CD4+INF-γ+ (Th1) cells, as the cellular mediators of inflammation were significantly decreased, while CD4+FoxP3+ cells (Treg) as the mediators of immunomodulation were significantly increased. These findings are consistent with previous studies, which reported a decrement in Th1/Th17, but an increment in Treg response following MSCs transplantation. The abovementioned changes could extend the skin graft survival by inhibiting different graft rejection mechanisms. So, histopathological reports in a short time was (acute phase) confirmed the immunomodulatory properties of MSCs after skin transplantation *in vivo* that already we had shown *in vitro*. However, further research with more examples in a long time (chronic phase) is needed in future studies. 

## Conclusion

Our study describes immunomodulatory effect of hADMSCs and hBM-MSCs transplantation on monkey T cells subsets, 96 hours after allogenic skin graft, in a monkey model; nevertheless, due to research limitations, as our findings are limited to a small sample size and the acute phase of immune response following skin graft, longer *in vivo* experiments are required to get more detailed information on the chronic phase of immune response. 

## Supplementary PDF


