# Supplementary material for: Immunomodulatory Activity of Human Bone Marrow and Adipose-Derived Mesenchymal Stem Cells Prolongs Allogenic Skin Graft Survival in Nonhuman Primates
Source: Cell J. 2021 Mar 1;23(1):1–13. doi: 10.22074/cellj.2021.6895 (PMC7944119; doi:10.22074/cellj.2021.6895)
Supplement: Supplementary file 1 [file Cell-J-23-1-s01.pdf]

www.celljournal.org

Supplementary Information for

Immunomodulatory Activity of Human Bone Marrow and Adipose-Derived Mesenchymal Stem Cells Prolongs Allogenic Skin Graft Survival in Nonhuman Primates

Fattah Sotoodehnejadnematalahi, Ph.D.[1#], Reza Moghadasali, Ph.D.[2, 3, 4#*], Mostafa Hajinasrollah, D.V.M.[2], Ehsan Ehsani, Ph.D.[2, 5], Ensiyeh Hajizadeh-Saffar, M.D., Ph.D.3, Niloofar Sodeifi, M.D.[6], Reza F. Saidi, M.D.[7], Morteza Zarrabi, Ph.D.[8], Mohammad Farzanehkhah, M.Sc.[8], Bahareh Sadeghi, M.Sc.[8], Hossein Baharvand, Ph.D.[1, 4], Nasser Aghdami, M.D., Ph.D.[4]

1. Department of Biology, School of Basic Science, Science and Research Branch, Islamic Azad University, Tehran, Iran
2. Department of Stem Cells and Developmental Biology, Cell Sciences Research Center, Royan Institute for Stem Cell Biology and Technology, ACECR, Tehran, Iran
3. Department of Regenerative Medicine, Cell Sciences Research Center, Royan Institute for Stem Cell Biology and Technology, ACECR, Tehran, Iran
4. Department of Developmental Biology, University of Science and Culture, Tehran, Iran
5. Department of Biology, Roudehen Branch, Islamic Azad University, Roudehen, Iran
6. Department of Andrology, Reproductive Biomedicine Research Center, Royan Institute for Reproductive Biomedicine, ACECR, Tehran, Iran
7. Department of Surgery, Shariati Hospital, Tehran University of Medical Sciences, Tehran, Iran
8. Royan Stem Cell Technology Company, Cord Blood Bank, Tehran, Iran

#The first two authors equally contributed to this work.

**Corresponding Address: P.O.Box: 16635-148, Department of Stem Cells and Developmental Biology, Cell Sciences Research Center, Royan Institute for Stem Cell Biology and Technology, ACECR, Tehran, Iran*
Email: rezamoghadasali@royaninstitute.org

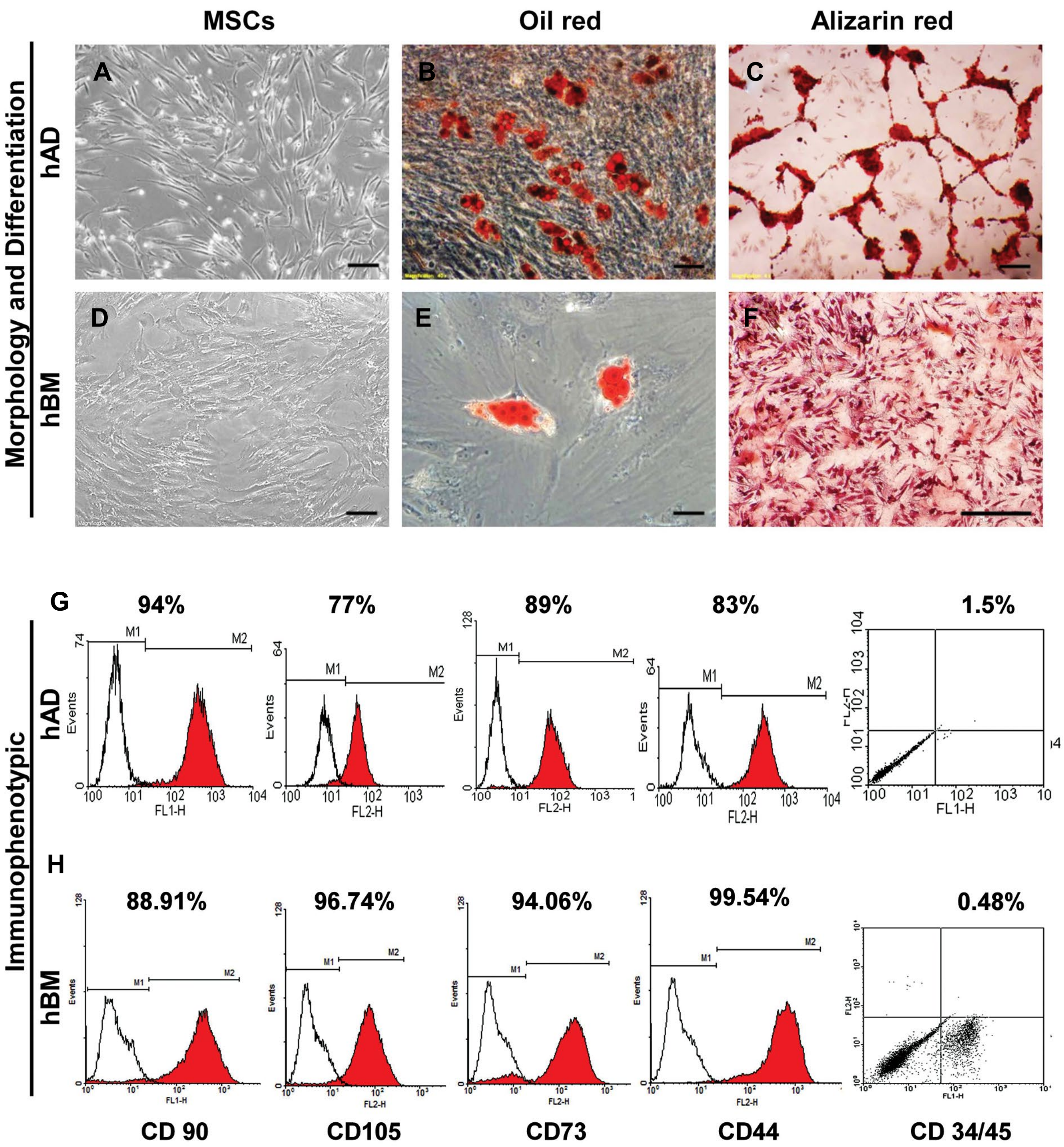

Fig.S1: Characterization of hAD-MSCs and hBM-MSCs. For the assessment of differentiation capacity, hAD-MSCs and BM-MSCs were differentiated into adipocyte and osteocyte lineages in specific induction media. Oil red and alizarin red dye were used to examine adipogenic and osteogenic differentiation capacity, respectively. Immunophenotypic characterization of hAD-MSCs and hBM-MSCs was performed by a flow cytometer. MSCs (hAD vs. hBM) were positive for CD44 (83 vs. 99.54%, respectively), CD73 (89 vs. 94.06%, respectively), CD90 (94 vs. 88.91%, respectively) and CD105 (77 vs. 96.74%, respectively), as mesenchymal stem cell markers. The results showed that hAD-MSCs and hBM-MSCs were not contaminated by hematopoietic cell lineages (i.e., cells were CD34 and CD45 negative).
hAD-MSCs; Human adipose-derived mesenchymal stem cells, and hBM-MSCs; Human bone marrow-derived mesenchymal stem cells.
